# Supplementary material for: Wide field light-sheet microscopy with lens-axicon controlled two-photon Bessel beam illumination
Source: Nat Commun. 2021 May 20;12:2979. doi: 10.1038/s41467-021-23249-y (PMC8137944; doi:10.1038/s41467-021-23249-y)
Supplement: Supplementary file 1 — Supplementary Information [file 41467_2021_23249_MOESM1_ESM.pdf]

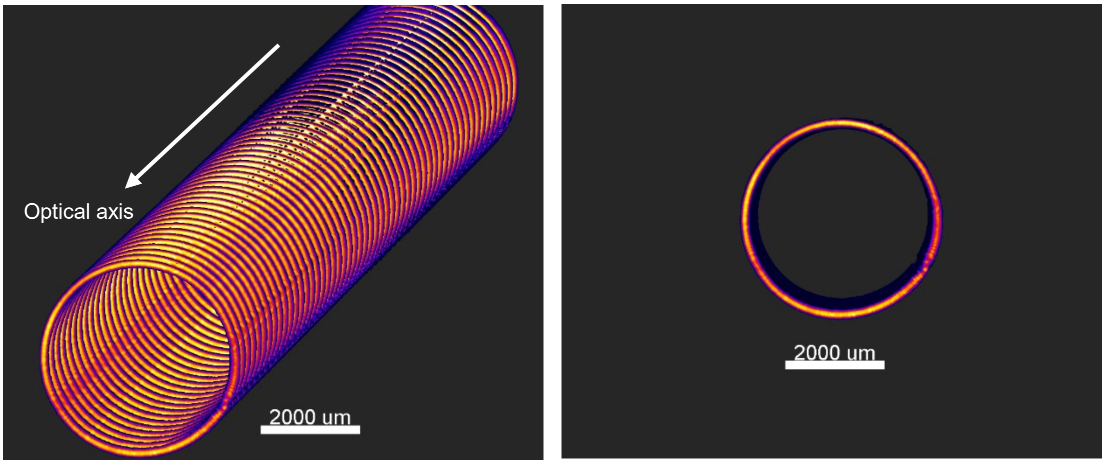

Supplementary Figure 1. 3D Reconstructed image of annular illumination patterns at the back focal plane of IO lens. Images are acquired with 0.5 mm step plus/minus 10 mm from the back focal plane. Left: oblique view, right: transverse view.

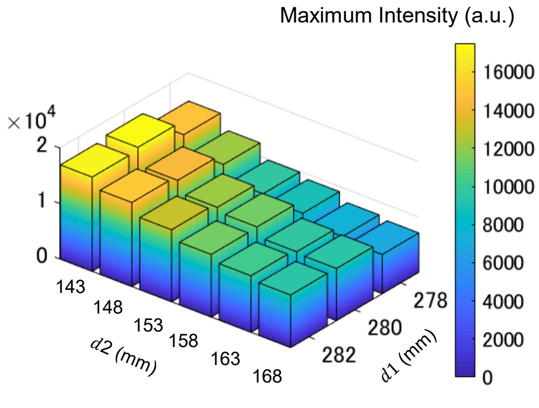

Supplementary Figure 2. Maximum intensities of the Bessel beam profiles. a.u., arbitrary unit.

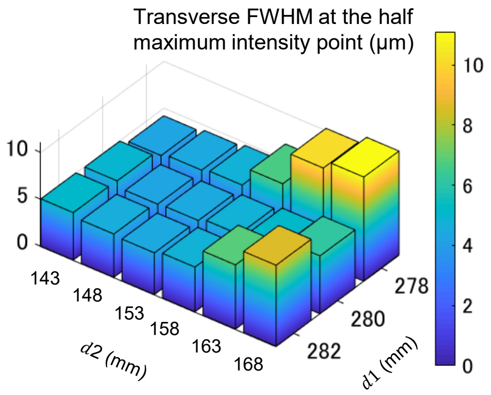

Supplementary Figure 3. Transverse extent at the half maximum point of fluorescent intensity for Bessel beam profiles.

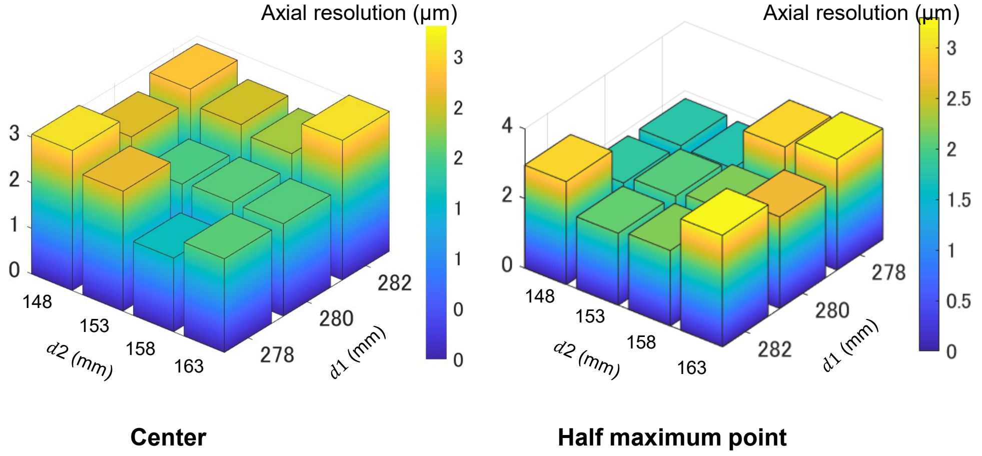

Supplementary Figure 4. PSF values based on the fluorescent beads measurements for different d_1 and d_2 Bessel beam parameters. Left calculated from image center, right calculated from the half maximum intensity point.

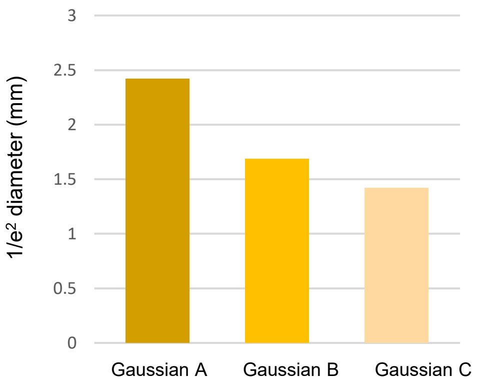

Supplementary Figure 5. Transverse $1/e^2$ diameter of the Gaussian beam at the back focal plane of the IO lens.

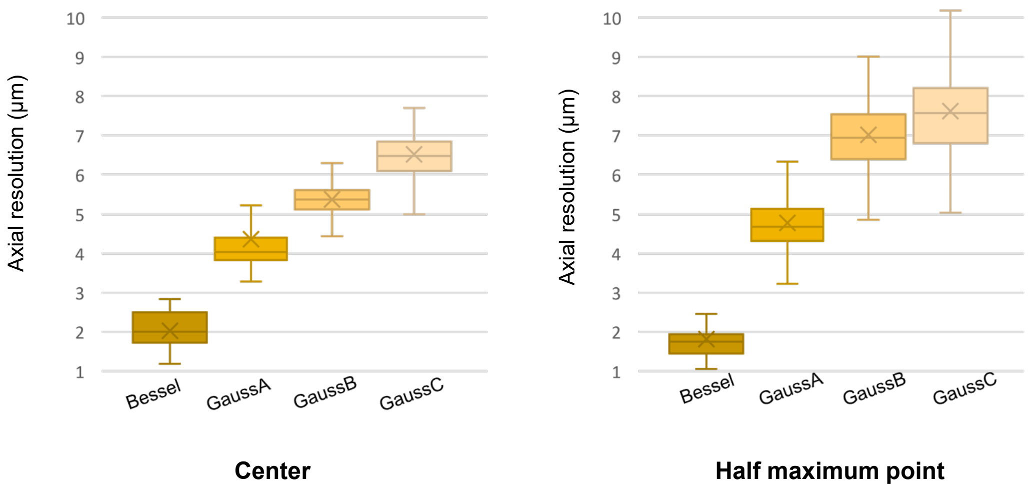

Supplementary Figure 6. PSF values based on the fluorescent bead measurements for the Bessel and three different Gaussian beams. (left) Calculated from the image center. (right) Calculated from the half maximum intensity point. Lines of the box indicate the first quantile, median, and third quantile. Whiskers of the lower and upper indicate the minimum and maximum, respectively. Cross mark represents the average. The PSF calculation employed over thirty independent beads as data points.

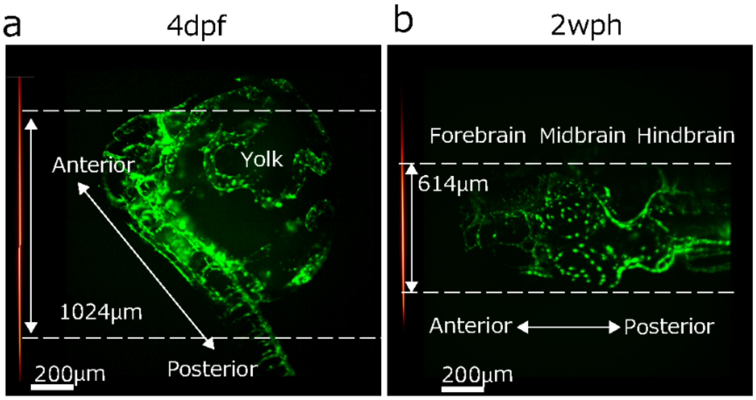

Bessel
d1=280mm, *d2*=163mm

Bessel
d1=280mm, *d2*=143mm

Supplementary Figure 7. Comparison of FLT4-EGFP strain embryo and larva with different Bessel beams. (a) Maximum intensity projection view over entire volume of a 4 dpf embryo of the FLT4-EGFP strain, which expresses EGFP in the lymphatic endothelial cells acquired with a Bessel beam of longitudinal extent 1024 μm (d_1 =280, $d_2 = 163$). White dashed lines indicate the half maximum intensity point of the Bessel beam. (b) Maximum intensity projection view over the entire volume of a 2 wph FLT4-EGFP strain larva acquired with a Bessel beam of longitudinal extent 614 μm (d_1 =280, $d_2 = 143$). Exposure time, *z*-interval, and *xy* are 300 ms, 2 μm, and 1024×1024 pixels, respectively. (a,b) n=3 biologically independent larvae are subjected to the analysis.

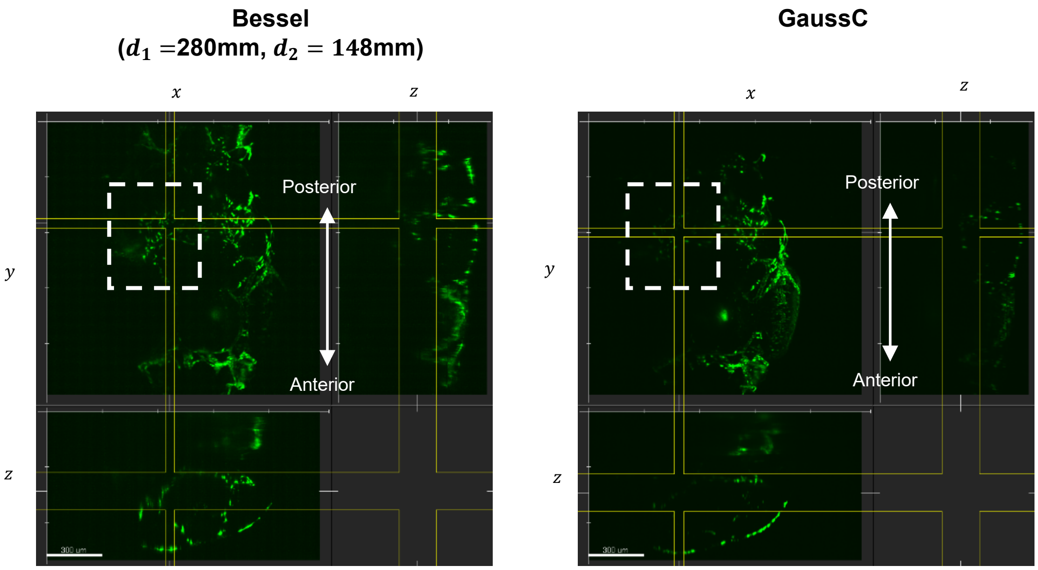

Supplementary Figure 8. Comparison of FLT4-EGFP strain imaging for Bessel and Gaussian C beams. Widths of sliced views are defined by the yellow lines. Laser beams are injected from the right side of the *xy* plane. White dashed boxes indicate the distal areas of the laser incidence where significant differences appear in the comparison of Gaussian and Bessel beam irradiations. n=3 biologically independent larvae are subjected to the analysis.

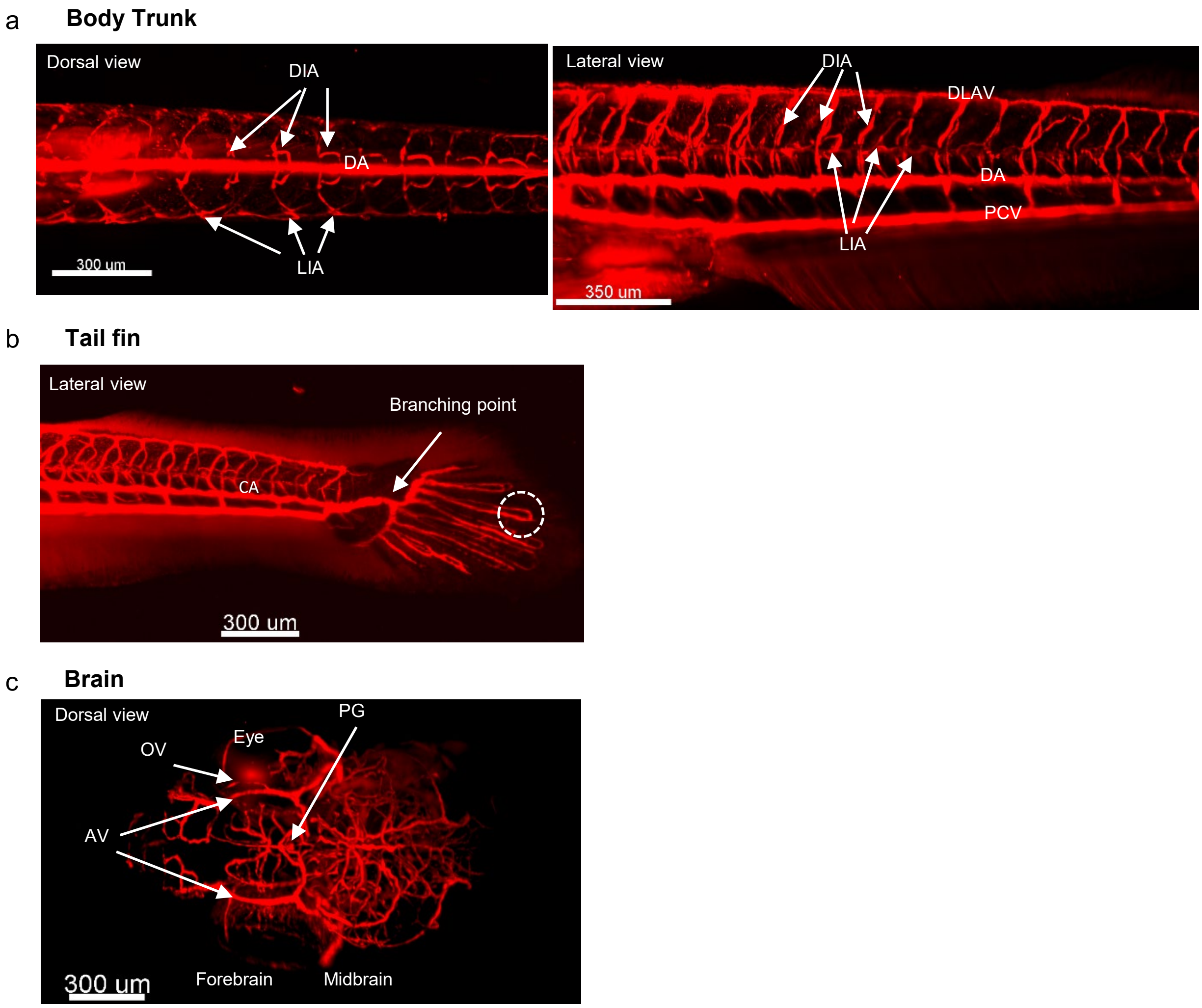

Supplementary Figure 9. Anatomical structures of blood vessels in organs. (a) Body trunk. (left) Viewed from the dorsal side. (right) Viewed from the lateral side. (b) Tail fin viewed from the lateral side. (c) Brain viewed from the dorsal side. (a-c) n=3 biologically independent larvae are subjected to the analysis. Abbreviations; DA, dorsal aorta; DIA, dorsal intersegmental aorta; LIA lateral intersegmental aorta; PCV, posterior cardinal vein; DLAV, dorsal longitudinal anastomotic vessel; CA, caudal aorta; PG, pineal gland; OV, ophthalmic vessel; AV, anterior vein.

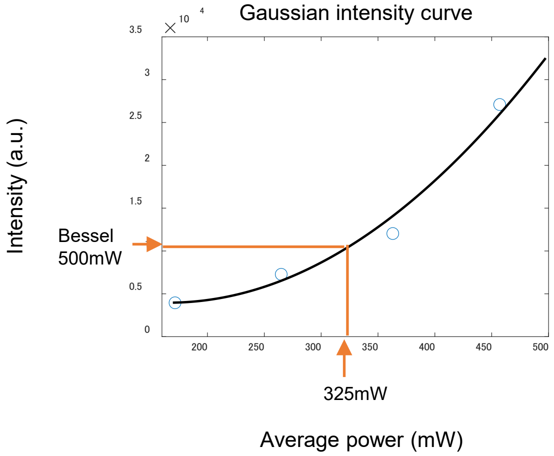

Supplementary Figure 10. Determination of the average laser power of Gaussian C setups which generate equal fluorescent intensities of a Bessel beam ($d_1 = 280\text{mm}, d_2 = 148\text{mm}$) at 500 mW. The average laser power is measured at the back focal plane of the IO lens. Two photon excited fluorescence are measured using 2-μm diameter green-yellow beads. For calculations of bead intensities, the cropped images of 256×1024 pixels are created. Using maximum intensity projection view of the *z*-stack images, the centroid intensities of the beads are measured and the average values are plotted as blue circles. The intensity curve is fitted by a quadratic function of average laser power. a.u., arbitrary unit.

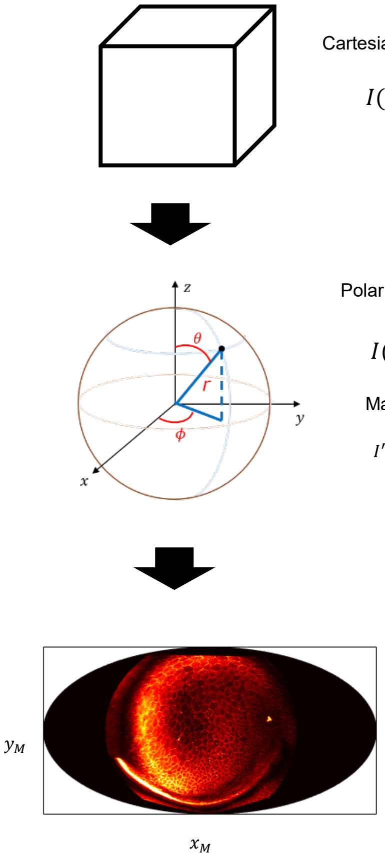

Cartesian coordinates

$$I(x, y, z; t)$$

Polar coordinates

$$I(r, \theta, \phi; t)$$

Maximum intensity Projection

$$I'(\theta, \phi; t) = \max_{r \sim r+\Delta r} I(r, \theta, \phi; t)$$

Mollweide Projection

$$I'(x_M, y_M; t)$$

$$x_M = \frac{2}{\pi}(\phi - \pi)\cos\alpha$$

$$y_M = \sin\alpha$$

$$2\alpha + \sin 2\alpha = \pi \sin(\theta - \pi)$$

Supplementary Figure 11. Processes of the Mollweide projection.

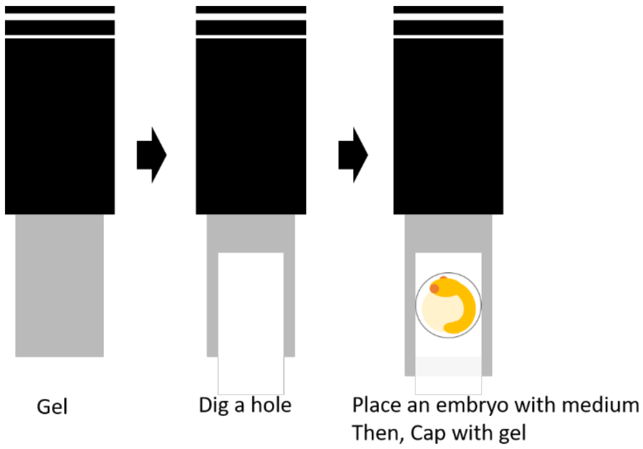

Supplementary Figure 12. Gel cage sample mounting method.
