# Supplementary material for: Wide field light-sheet microscopy with lens-axicon controlled two-photon Bessel beam illumination
Source: Nat Commun. 2021 May 20;12:2979. doi: 10.1038/s41467-021-23249-y (PMC8137944; doi:10.1038/s41467-021-23249-y)
Supplement: Supplementary file 3 — Description of Additional Supplementary Files [file 41467_2021_23249_MOESM3_ESM.pdf]

Description of Additional Supplementary Files

Title: Supplementary Movie 1
Description: Time course imaging of a FLT4-EGFP Medaka during 80–155 hpf in the dorsal view.

Title: Supplementary Movie 2
Description: Time course imaging of a FLT4-GFP Medaka during 80–155 hpf in the lateral view.

Title: Supplementary Movie 3
Description: Ca^{2+} imaging of a Medaka embryo with GCaMP6f.

Title: Supplementary Code
Description: The MATLAB program package that performs a numerical integration of the lens-axicon triplet formula.
