# Supplementary material for: Wide field light-sheet microscopy with lens-axicon controlled two-photon Bessel beam illumination
Source: Nat Commun. 2021 May 20;12:2979. doi: 10.1038/s41467-021-23249-y (PMC8137944; doi:10.1038/s41467-021-23249-y)
Supplement: Supplementary file 8 — Reporting Summary [file 41467_2021_23249_MOESM8_ESM.pdf]

nature research

Corresponding author(s): Takashi Saitou

Last updated by author(s): Mar 24, 2021

Reporting Summary

Nature Research wishes to improve the reproducibility of the work that we publish. This form provides structure for consistency and transparency in reporting. For further information on Nature Research policies, see our Editorial Policies and the Editorial Policy Checklist.

Statistics

For all statistical analyses, confirm that the following items are present in the figure legend, table legend, main text, or Methods section.

n/a	Confirmed	
☐	☒	The exact sample size (*n*) for each experimental group/condition, given as a discrete number and unit of measurement
☐	☒	A statement on whether measurements were taken from distinct samples or whether the same sample was measured repeatedly
☐	☒	The statistical test(s) used AND whether they are one- or two-sided<br>*Only common tests should be described solely by name; describe more complex techniques in the Methods section.*
☒	☐	A description of all covariates tested
☐	☒	A description of any assumptions or corrections, such as tests of normality and adjustment for multiple comparisons
☐	☒	A full description of the statistical parameters including central tendency (e.g. means) or other basic estimates (e.g. regression coefficient) AND variation (e.g. standard deviation) or associated estimates of uncertainty (e.g. confidence intervals)
☐	☒	For null hypothesis testing, the test statistic (e.g. *F*, *t*, *r*) with confidence intervals, effect sizes, degrees of freedom and *P* value noted<br>*Give P values as exact values whenever suitable.*
☒	☐	For Bayesian analysis, information on the choice of priors and Markov chain Monte Carlo settings
☒	☐	For hierarchical and complex designs, identification of the appropriate level for tests and full reporting of outcomes
☒	☐	Estimates of effect sizes (e.g. Cohen's *d*, Pearson's *r*), indicating how they were calculated

Our web collection on statistics for biologists contains articles on many of the points above.

Software and code

Policy information about availability of computer code

Data collection	Labview2015 (National Instruments), Beam Gage Professional 6.12 (Ophir-Spiricon)
Data analysis	PSFj(http://www.knoplab.de/psfj/), Imaris 8.4.0 (Bitplane), Fiji (https://imagej.net/Fiji), Excel2019(Microsoft), MATLAB R2018b and later (Mathworks), the MATLAB program package that performs a numerical integration of the lens-axicon triplet formula is provided as a Supplementary Code.

For manuscripts utilizing custom algorithms or software that are central to the research but not yet described in published literature, software must be made available to editors and reviewers. We strongly encourage code deposition in a community repository (e.g. GitHub). See the Nature Research guidelines for submitting code & software for further information.

Data

Policy information about availability of data

All manuscripts must include a data availability statement. This statement should provide the following information, where applicable:

- Accession codes, unique identifiers, or web links for publicly available datasets
- A list of figures that have associated raw data
- A description of any restrictions on data availability

The datasets generated during and/or analysed during the current study are available in the figshare repository https://doi.org/10.6084/m9.figshare.14229224. The source data underlying Figs. 2c,2f-h, 3a-d, 4b-f, and 8c, and Supplementary Figs. 2, 3, 4, 5, 6 10 are provided as a Supplementary Data.

Field-specific reporting

Please select the one below that is the best fit for your research. If you are not sure, read the appropriate sections before making your selection.

☒ Life sciences ☐ Behavioural & social sciences ☐ Ecological, evolutionary & environmental sciences

For a reference copy of the document with all sections, see nature.com/documents/nr-reporting-summary-flat.pdf

Life sciences study design

All studies must disclose on these points even when the disclosure is negative.

Sample size	Number of medaka samples used: FLT4-EGFP ×3 for whole body imaging of the lymphatic and blood vessels, KIF5Aa-GFP ×5 for self-reconstruction assessment, beta-actin-DsRed2 ×3 for each different beam condition of phototoxicity experiment, and FLT4-EGFP ×2 for time-lapse analysis of embryonic development, STII ×1 for Ca2+ imaging. Sample size was determined with considering reproducibility of the experimental results. The repeated data were consistent, thus these provided reproducible dataset. For Ca2+ imaging, single sample was used because this is just for fast imaging demonstration.
Data exclusions	No data were excluded.
Replication	For reproducibility of phototoxicity and self-reconstruction evaluations, we measured samples (KIF5Aa-GFP ×5 and beta-actin-DsRed2 ×3). These data are consistent and we consider that replications may be successful.
Randomization	This is not relevant to our study. All medaka strains were maintained in freshwater tanks with same conditions described in the text.
Blinding	The samples were blindly chosen from a pool of embryos and larvae. For quantitative data analysis, all measured samples were analyzed.

Reporting for specific materials, systems and methods

We require information from authors about some types of materials, experimental systems and methods used in many studies. Here, indicate whether each material, system or method listed is relevant to your study. If you are not sure if a list item applies to your research, read the appropriate section before selecting a response.

Materials & experimental systems

n/a	Involved in the study	
☒	☐	Antibodies
☒	☐	Eukaryotic cell lines
☒	☐	Palaeontology and archaeology
☐	☒	Animals and other organisms
☒	☐	Human research participants
☒	☐	Clinical data
☒	☐	Dual use research of concern

Methods

n/a	Involved in the study	
☒	☐	ChIP-seq
☒	☐	Flow cytometry
☒	☐	MRI-based neuroimaging

Animals and other organisms

Policy information about studies involving animals; ARRIVE guidelines recommended for reporting animal research

Laboratory animals	The Medaka strain, See-Through II (STII) strain (StrainID: MT112), Tg(pKIF5Aa-GFP) (StrainID: TG1156), d-rR-Tg(beta-actin-loxP-DsRed2-loxP-GFP) (StrainID: TG861) supplied by NBRP Medaka (https://shigen.nig.ac.jp/medaka/), and the transgenic strain FLT4-EGFP. The animals used for the experiments include male and female, from embryos (1-6dph) to juveniles (2-4wph).
Wild animals	The study did not involve wild animals.
Field-collected samples	The study did not involve samples collected from the field.
Ethics oversight	All experiments were conducted in accordance with the guidelines of the ethics committee for animal experiments of Ehime University.

Note that full information on the approval of the study protocol must also be provided in the manuscript.
